# Impact of Age and Sex on Outcomes and Hospital Cost of Acute Asthma in the United States, 2011-2012

**DOI:** 10.1371/journal.pone.0157301

**Published:** 2016-06-13

**Authors:** Joe G. Zein, Belinda L. Udeh, W. Gerald Teague, Siran M. Koroukian, Nicholas K. Schlitz, Eugene R. Bleecker, William B. Busse, William J. Calhoun, Mario Castro, Suzy A. Comhair, Anne M. Fitzpatrick, Elliot Israel, Sally E. Wenzel, Fernando Holguin, Benjamin M. Gaston, Serpil C. Erzurum, The Severe Asthma Research Program

**Affiliations:** 1 Department of Pathobiology, Lerner Research Institute, and Respiratory Institute, Cleveland Clinic, Cleveland, Ohio, United States of America; 2 Department of Outcomes Research, Anesthesiology Institute, Cleveland Clinic, Cleveland, Ohio, United States of America; 3 Department of Pediatrics, University of Virginia School of Medicine, Charlottesville, Virginia, United States of America; 4 Department of Epidemiology and Biostatistics, Case Western Reserve University, Cleveland, Ohio, United States of America; 5 Center for Genomics and Personalized Medicine, Wake Forest University School of Medicine, Winston-Salem, North Carolina, United States of America; 6 Department of Medicine, the University of Wisconsin, School of Medicine and Public Health, Madison, Wisconsin, United States of America; 7 Department of Medicine, University of Texas Medical Branch, Galveston, Texas, United States of America; 8 Department of Medicine, Washington University School of Medicine, St. Louis, Missouri, United States of America; 9 Department of Pediatrics, Emory University School of Medicine, Atlanta, Georgia, United States of America; 10 Pulmonary Division, Harvard Medical School, Brigham and Women’s Hospital, Boston, Massachusetts, United States of America; 11 The Asthma Institute, the University of Pittsburgh, Pittsburgh, Pennsylvania, United States of America; 12 Department of Pediatric, Rainbow Babies & Children’s Hospital, Cleveland, Ohio, United States of America; University of Athens, GREECE

## Abstract

**Background:**

Worldwide, asthma is a leading cause of morbidity, mortality and economic burden, with significant gender and racial disparities. However, little attention has been given to the independent role of age on lifetime asthma severity and hospitalization. We aimed to assess the effect of age, gender, race and ethnicity on indicators of asthma severity including asthma related hospitalization, mortality, hospital cost, and the rate of respiratory failure.

**Methods:**

We analyzed the 2011 and 2012 Healthcare Cost and Utilization Project- National Inpatient Sample (NIS). We validated and extended those results using the National Heart, Lung, and Blood Institute-Severe Asthma Research Program (SARP; 2002–2011) database. Severe asthma was prospectively defined using the stringent American Thoracic Society (ATS) definition.

**Results:**

Hospitalization for asthma was reported in 372,685 encounters in 2012 and 368,528 in 2011. The yearly aggregate cost exceeded $2 billion. There were distinct bimodal distributions for hospitalization age, with an initial peak at 5 years and a second at 50 years. Likewise, this bimodal age distribution of patients with severe asthma was identified using SARP. Males comprised the majority of individuals in the first peak, but women in the second. Aggregate hospital cost mirrored the bimodal peak distribution. The probability of respiratory failure increased with age until the age of 60, after which it continued to increase in men, but not in women.

**Conclusions:**

Severe asthma is primarily a disease of young boys and middle age women. Greater understanding of the biology of lung aging and influence of sex hormones will allow us to plan for targeted interventions during these times in order to reduce the personal and societal burdens of asthma.

## Introduction

Asthma is a chronic health condition associated with significant health and economic burden to patients, their families, and society. It affects 25.7 million people; 7.0 million children under the age of 18 years. The incidence of asthma is increasing. In 1980 it was 3.6%, increasing to 8.2% by 2009 [1, 2]. This increased asthma incidence is of particular concern given that asthma is a significant cause of morbidity and utilization of healthcare resources across the lifespan. In 2010, asthma resulted in 1.8 million emergency department (ED) visits and 439,000 asthma-related hospitalizations.

Severe asthma accounts for the majority of healthcare costs due to hospitalizations and ED visits, and is associated with the highest asthma related mortality [3]. While asthma is the leading cause of ED visits and the third cause of hospitalization in children aged 1 to 17 years [4, 5], data obtained from the Severe Asthma Research Program (SARP) [6] identified that severe asthma patients were older than patients with mild to moderate asthma [6]. Even after adjustment for many age-related comorbidities, older asthmatics were 2.7 times more likely to have “severe asthma” as compared to young adult asthma patients [7]. Gender difference in asthma incidence, prevalence and severity are also seen [1, 2]. Asthma is more prevalent in boys 4 to 14 years compared to girls (11.5 vs. 9.9%). However, after puberty, asthma becomes more prevalent and severe in women [8]. Interestingly, a reversal of the gender switch occurs with menopause, when asthma becomes once again more severe in older male adults [7]. While the distribution of asthma prevalence is uniform across the ages, little is known regarding the influence of age and gender on the prevalence of severe asthma in these patients[8].

Here, we assessed the effect of age and its influence by gender and race on indicators of asthma severity such as asthma related hospitalization and cost, hospital mortality, and the rate of respiratory failure in two administrative databases NIS 2011 and 2012. We compared these results with age and gender-dependent risk of a well characterized severe asthma cohort using the National Heart, Lung, and Blood Institute- Severe Asthma Research Program.

## Methods

In this study, data was abstracted from the 2011 and 2012 Nationwide Inpatient Sample (NIS). The NIS is included in the Healthcare Cost and Utilization Project (HCUP) family. It is created by the Agency for Healthcare Research and Quality's (AHRQ) through a Federal-State-Industry partnership. The database is a 20% weighted sample of all United States (US) hospital discharges, constituting more than 7 million discharges annually. A data user agreement was signed with the Agency for Healthcare Research and Quality's (AHRQ).

### Framework and Study Population

To analyze the impact of aging on asthma severity, adjustments were made for age related comorbidities, gender and race which are known to impact asthma severity [9, 10] [S1 Fig]. In each visit, up to 25 diagnoses are reported in NIS using the International Classification of Disease, ninth revision- Clinical Modification (ICD9_CM codes). DX1, reflects the principal (primary) admitting diagnosis. DX2 to DX25 reflect the 24 reported secondary diagnoses in NIS. The NIS Severity Measures File list up to 29 comorbid conditions for each visit [S1 Table].

The ICD9-CM codes of 493.xx were used to identify patients admitted with a principal diagnosis (DX1) of asthma [S1 Appendix] [11]. Patients were excluded if they had a secondary diagnosis of chronic obstructive pulmonary disease (COPD), bronchiolitis with Respiratory Syncytial Virus (RSV), were active or past smokers, or if they had a chronic lung disease diagnosed as a comorbidity [S1 Appendix and S2 Fig]. Additionally, infants were excluded due to the frequent misdiagnosis of asthma in infants with wheezing. The impact of these patient group exclusions were tested with sensitivity analysis. Moreover, we cross-checked our HCUP analysis using extensively and precisely characterized asthma in the National Heart, Lung, and Blood Institute (NHLBI) Severe Asthma Research program (SARP) between 2002 to 2011. A written informed consent was obtained from all adults, or from the parents of minor participants. The project and the informed consent were approved by the Institutional Review Board at all 10 SARP sites enrolling participants [6], which includes the Cleveland Clinic Institutional Review Board (IRB), Cleveland, OH; Case Western Reserve University IRB, Cleveland, OH; the IRB at Wake Forest University, Winston-Salem, NC; the IRB for health Sciences Research at the University of Virginia, Charlottesville, VA, the IRB at the University of Wisconsin, Madison, WI; University of Pittsburgh IRB, Pittsburgh, PA; The Human Research Protection Office (HRPO) at the Washington University, St. Louis, MO; Partners Human Research Committee at the Brigham and Women’s Hospital, Boston MA; the University of Texas Medical Branch IRB, Galveston, TX; Emory IRB, at Emory University School of Medicine, Atlanta, GA; and the Imperial College Research Ethics Committee (ICREC), at the National Heart and Lung Institute, Imperial College, London, UK. To be enrolled in the study, all adult participants provided a written informed consent. The informed consent as well as the study protocol were approved by all local IRBs.

### Hospitalization and Outcome Variables

NIS includes variables; diagnosis, weekend admission, month of admission, payment source, urban vs rural location of patients’ residence, national quartiles for median household income by ZIP code, hospital geographic region (Northeast, Midwest, South and West), hospital length of stay, hospital disposition, and hospital charges. Using this data and applying cost to charge ratios, hospital mortality, respiratory failure, and services rendered and related costs were identified.

### Analysis

Using bootstrapping, unadjusted comparisons for continuous variables were analyzed. Two-sided p values <0.05 were considered significant. National estimates were produced by applying the “weight" variable to the data. All other analyses were performed using the unweighted NIS asthma data. To test the effect of aging on mortality and respiratory failure, we fitted a logistic regression model. The initial model included age, gender, race, history of gastro-esophageal reflux disease, quartiles for median household income, admission month, weekend admission, payer information, and a modified Elixhauser comorbidity index[12, 13]. The Elixhauser comorbidity index summarizes disease burden and predicts hospital mortality. The final model was used to plot the curve of the risk adjusted probability of hospital mortality and respiratory failure. A restricted cubic spline model with 3 knots was fitted to better describe the nonlinear relationship between age and outcomes measures such as mortality and respiratory failure [14]. The descriptive statistics and the logistic regression were conducted using R Core Team (2015). R: A language and environment for statistical computing. R Foundation for Statistical Computing, Vienna, Austria

### Sensitivity analyses

Although the fitted logistic regressions ensured variable selection decisions, the results needed to be validated with new data. Therefore, we used the 2012 databases to build the models, and the 2011 databases for validation. Importantly, in order to externally validate the reproducibility of those results, we used SARP I&II databases. SARP enrolled participants between 2002 and 2011 and was designed to study severe asthma [6]. In contrast to HCUP where ICD9 codes were used to identify asthma, SARP prospectively collected a cohort where asthma severity (severe and non-severe asthma) was classified as defined by the American Thoracic Society [15]. In order to meet the definition of severe asthma, one or both major criteria and two minor criteria was required. The 2 major criteria include continuous or near continuous treatment with oral corticosteroids, or the need for high-dose inhaled corticosteroids therapy. The 7 minor criteria include the daily need for a controller medication in addition to inhaled corticosteroids, the daily need for short-acting beta-agonist use, persistent airway obstruction, one or more urgent care visits for asthma per year, three or more oral corticosteroid “bursts” per year, lung function deterioration with less than 25% reduction in oral or inhaled corticosteroid dose, and a previous history of near fatal asthma [15].

We anticipated two additional potential sources of bias. The first originating from the misdiagnosis of asthma in children younger than 1 year and the second resulting from mislabeling RSV bronchiolitis and COPD as asthma and vice versa. In order to account for such potential problems we analyzed the data initially after the exclusion of children younger than 1 year, and patients listed with COPD, RSV bronchiolitis or tobacco use as a secondary diagnosis, and subsequently compared those result to those obtain from the original data without any exclusions. Likewise, in order to reproduce those results, similar analysis was performed using the NIS 2011 database. Furthermore, the use of SARP obviated these biases, because active smokers and asthma patients with a smoking history that exceeded 5 pack-years were excluded. All SARP participants underwent lengthy testing in order to confirm asthma diagnosis, including spirometry and methacholine challenge testing. Children in SARP were diagnosed and characterized by pediatric asthma specialists.

## Results

### The bimodal distribution of asthma hospitalization

**Fig 1:**
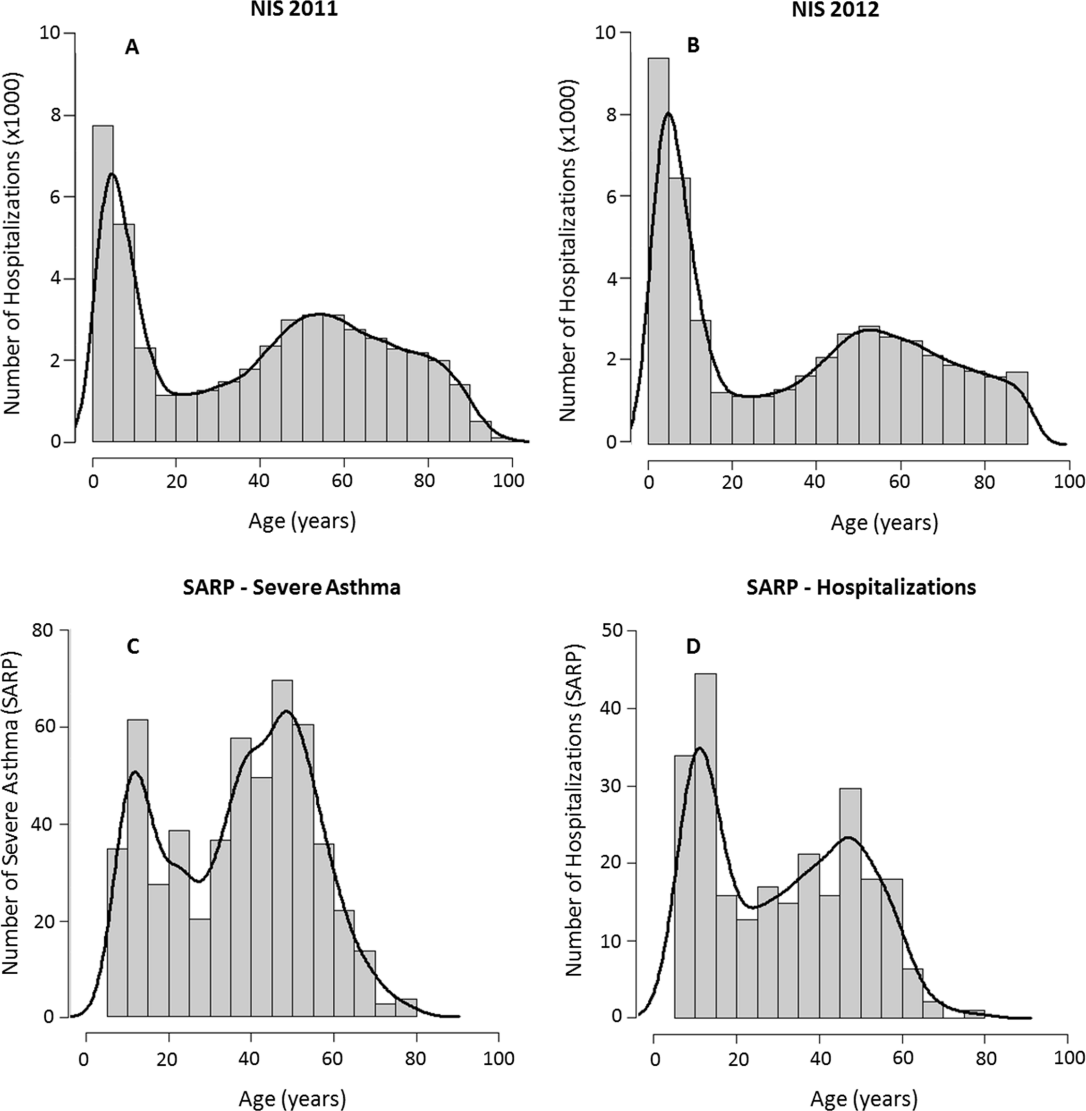
Histogram with smoothed density estimate of age distribution of asthma related hospitalizations in the United States in 2011 (Panel A) and 2012 (Panel B). Panel C and Panel D reflect the age distribution of severe asthma and asthma hospitalization in the Severe Asthma Research Program (SARP) database. All 3 databases show a bimodal age distribution of severe asthma.

Using ICD9-CM codes, we identified 79,810 asthma hospitalizations in the NIS 2011 and 74,110 in NIS 2012 database. National estimates produced by applying the “weight" variable to the initial data indicate that asthma resulted in 372,685 hospitalizations, 1,090 deaths (0.3%) with an aggregate cost of $ 2,231,112,607 in 2012 [S2 Table]. After excluding patients younger than 1 year and those with secondary diagnosis of COPD, smoking addiction, and RSV bronchiolitis, only 48,941 patients from NIS2011 and 45,670 from NIS 2012 were used for analysis [Table 1]. The average hospital length of stay was 3 days [Table 2]. Asthma hospitalization was higher in young children and in middle age adults [Fig 1]. This bimodal distribution was identical in NIS 2011 and NIS 2012 databases [Fig 1A and 1B].

**Table 1:** Demographics of patient hospitalized for asthma.

Characteristic		NIS 2011	NIS 2012	SARP
Number of encounters in the database		8,023,590	7,296,968	1,361
Number of asthma hospitalizations		48,941	45,670	237
Age (years)		40 [8,61]	35 [7, 60]	34 [24, 49]
Female gender (%)		30,703 (63.0)	27,228 (59.6)	141(59.5%)
Race (%)				
	White	20,263 (41.4)	18,730 (43.0)	98 (41)
	Black	13,658 (27.9)	13,891 (31.9)	114 (48)
	Hispanic	6,993 (14.3)	7,661 (17.6)	16 (17)
	Asian or Pacific Islander	924 (1.9)	1103 (2.5)	5 (2)
	Native American	359 (0.7)	314 (0.7)	0 (0)
	Others	1,791 (3.7)	1,827 (4.2)	6 (2)
Gastroesophageal reflux Disease (%)		8,247 (16.9)	6,853 (15.0)	90 (41)
Primary expected payer (%)				
	Medicare	14,825 (30.4)	12,026 (26.4)	
	Medicaid	15,138 (31.0)	16,346 (35.9)	
	Private including HMO	14,404 (29.5)	12,720 (27.9)	
	Self-pay	2,837 (5.8)	2,846 (6.2)	
	No charge	242 (0.5)	220 (0.5)	
	Other	1,369 (2.8)	1,420 (3.1)	
Median household income. (%)				
	$1–$38,999	17,509 (36.7)	17,050 (38.5)	
	$39,000–$47,999	11,230 (23.5)	10,643 (24.1)	
	$48,000–62,999	10,841 (22.7)	9,064 (20.5)	
	$63,000 or more	8,158 (17.1)	7,496 (16.9)	

NIS indicates National Inpatient Sample; SARP, Severe Asthma Research Program

**Table 2:** Clinical outcome of patients hospitalized for asthma.

Outcome		NIS 2011	NIS 2012
Disposition of patient (%)			
	Discharged home	42267 (86.6)	40248 (88.1)
	Transfer to short-term hospital	455 (0.9)	401 (0.9)
	Other transfers	2145 (4.4)	1636 (3.6)
	Home health care	3174 (6.5)	2717 (5.9)
	Against medical advice	638 (1.3)	555 (1.2)
	Hospital deaths	149 (0.3)	129 (0.3)
Respiratory failure (%)		4172 (8.5)	4449 (9.7)
Hospital length of stay (days)*		2.00 [2.00, 4.00]	2.00 [1.00, 4.00]
Total hospital charges (US $)*		13131 [7685, 23138]	13397 [7817, 23597]
Total hospital cost (US $)*		4106 [2565, 6621]	4099 [2559, 6675]

NIS indicates National Inpatient Sample

### The role of gender and race on asthma related hospitalization and cost

**Fig 2:**
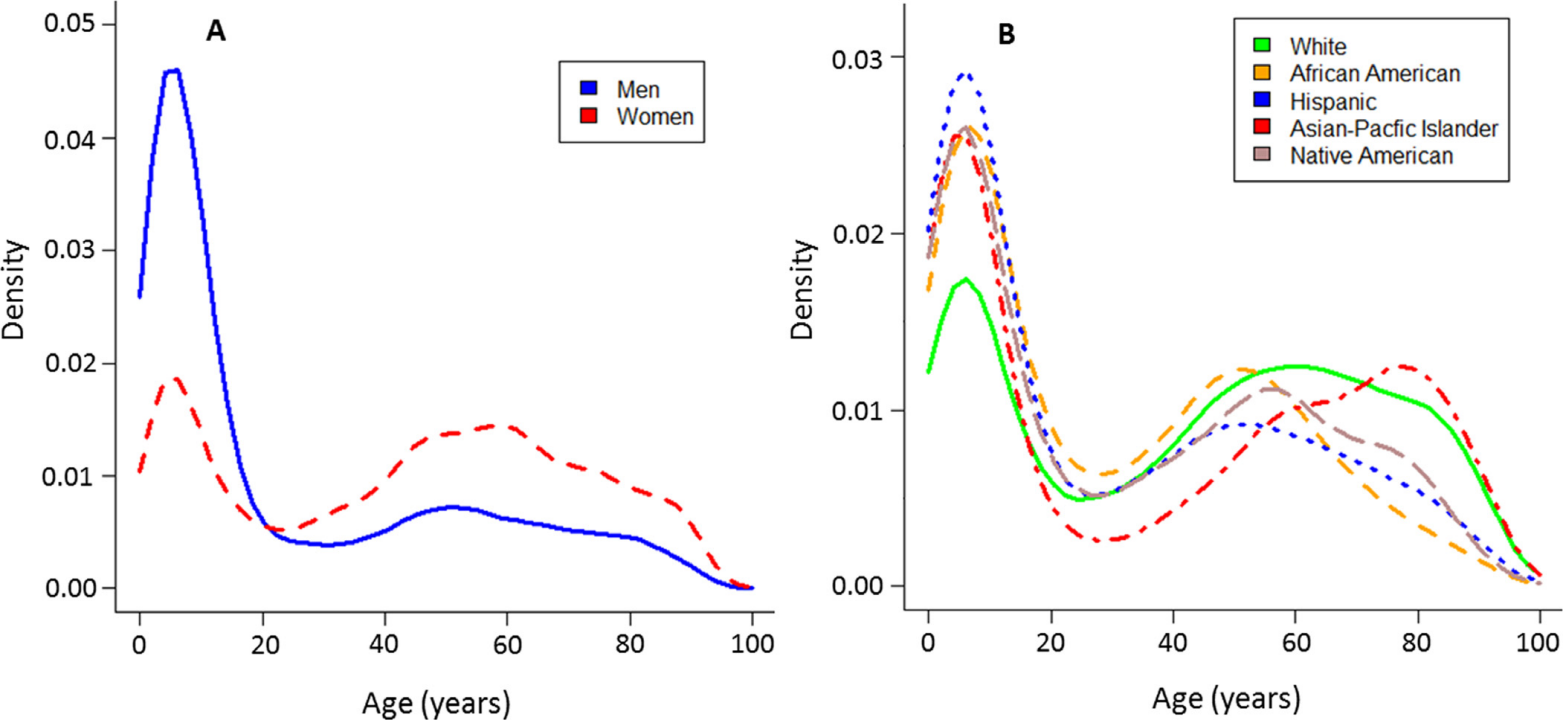
Density plots of the distribution of asthma hospitalizations stratified by gender and race. Panel A shows that asthma hospitalization is more frequent among young boys and middle age women. Panel B shows a bi-modal distribution of asthma severity across different races. Panel A and B are abstracted from NIS 2012.

Although, more hospitalizations were generally reported among women than men [Table 1], there were significant gender differences across the lifespan of asthmatics. Asthma related hospitalizations were most prevalent among young boys (age 1 to 10 years) and middle-aged women (age 40 to 60 years) [Fig 2A and S3 Fig]. This bimodal age-gender distribution was seen in 2011 and 2012 databases, and in SARP [S3 and S4 Figs], and remained evident among all races in the NIS populations. The age of onset of the second peak was impacted by race and ethnicity [Fig 2B]. Specifically, the age of hospitalization varied among race and ethnic background among adult asthmatics (age older than 18) hospitalized in 2012 from a mean of 51.5 years in African Americans to a mean age of 64.4 years in Asians (p<0.0001) [S3 Table].

### The role of age on asthma related respiratory failure and mortality

**Fig 3:**
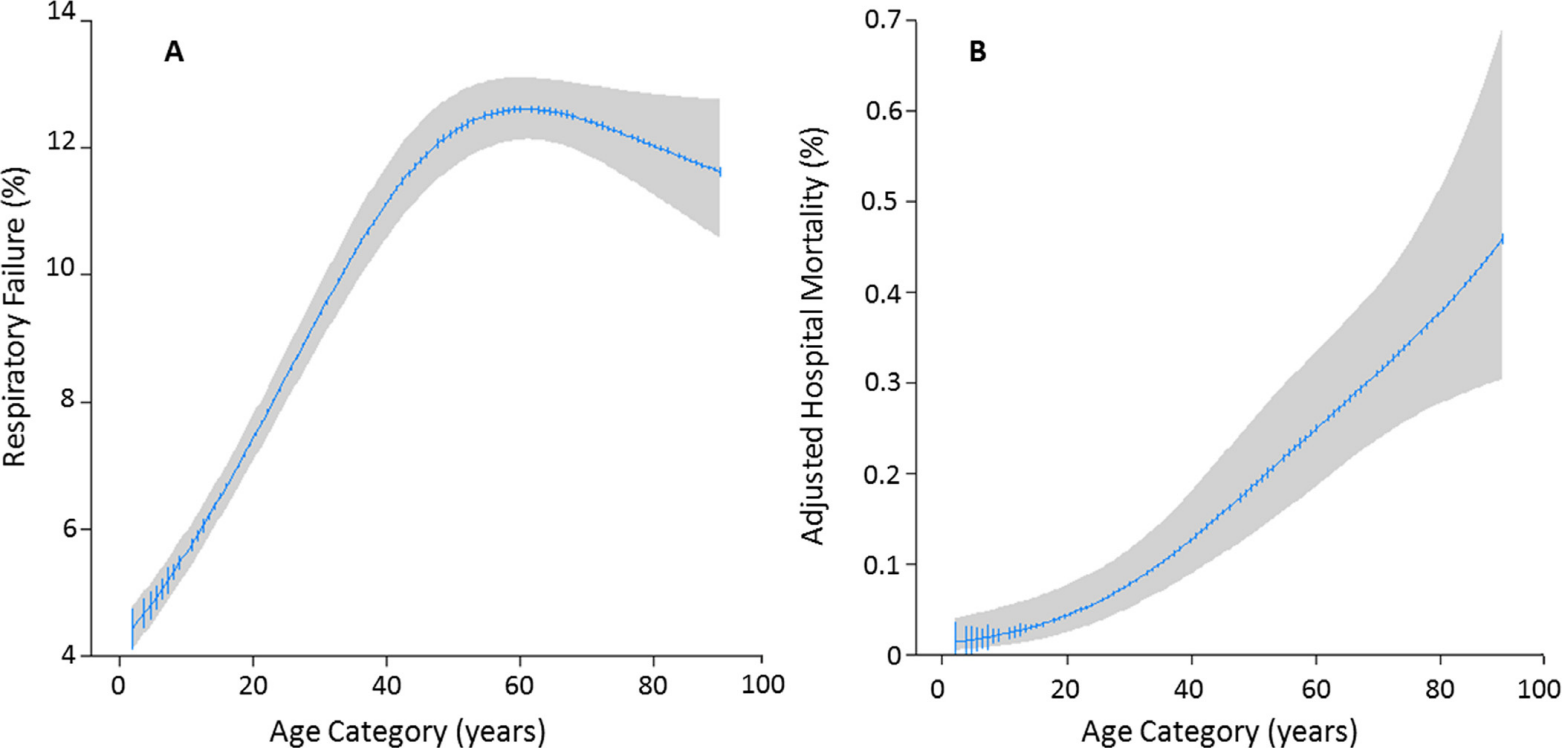
**Risk-adjusted probabilities of asthma related hospital mortality (Panel A) and respiratory failure (Panel B) as a function of age using the NIS 2012 database.** The probabilities were calculated by fitting a logistic regression using a restricted cubic spline function for age. The 95% CIs are indicated by the gray area around the fitted line.

Respiratory failure was reported in 4,172 (8.5%) and 4,449 (9.7%) patients hospitalized for asthma in the 2011 and 2012 NIS databases respectively [Table 2]. The probability of risk adjusted hospital mortality and respiratory failure was plotted as a function of age [Fig 3]. The risk of respiratory failure increased by 34% [adjusted OR (95CI) 1.34 (1.30–1.39)] for each 10 years increment in age until the age of 60 years. Afterward the risk did not significantly increase [adjusted OR (95CI) 0.98 (0.95–1.02)]. This plateau in risk of respiratory failure after age of 60 was due to a lower probability of respiratory failure among women compared to men [S5 Fig].

Similar to the risk of respiratory failure, the adjusted probability of death increases by 47% [adjusted OR (95CI) 1.47 (1.31–1.66)] for each 10 years increment in age [Fig 3B]. While a higher number of older women died of asthma compared to men [S6A Fig], there were almost twice as many total women hospitalized for asthma. Therefore, the mortality rates among hospitalized men and women were not significantly different [OR (95CI) 1.36 (0.94–1.96) for women vs. men] [S6B Fig].

### Results from SARP

Among the 1,361 asthma patients enrolled in SARP, 583 patients met the American Thoracic Society definition of severe asthma and 237 patients had required hospitalization during the year prior to their visit. The SARP age distribution for severe asthma and hospitalization mirrored the bimodal distribution seen in NIS 2011 and NIS 2012 [Fig 1C and 1D]. The slight attenuation of the initial peak in SARP may reflect the fact that SARP did not enroll children younger than 6 years [Fig 1].

### The role of age on asthma related hospital cost

Hospitalization cost increased with age, with females having higher cost [S7 Fig and S4 Table]. The average hospitalization cost in 2012 was $6,875 for an asthma patient who did not develop respiratory failure. It was $14,839 for an asthmatic who developed respiratory failure and $29,043 for an individual requiring intubation and mechanical ventilation [expressed as Median (IQR) in S5 Table]. Aggregate hospital cost followed a bimodal distribution, similar to the bimodal peak of severe asthma, and was highest between the age of 1 to 10 years and after the age of 40 years [S7 Fig].

## Discussion

The principal finding of this study is that the age distribution of severe asthma and asthma hospitalization is bimodal, consistent with the hypothesis that asthma is more severe in young boys and middle age women. This bimodal distribution of severe asthma is in contrast with the age distribution of asthma prevalence which does not change appreciably across the ages[8]. The bimodal distribution of asthma severity was confirmed using SARP, a well characterized prospectively collected and strictly defined cohort of non-severe and severe asthmatic patients’ ages 6 to 80 years.

These findings strongly support gender differences in asthma severity throughout the life course. Asthma is more prevalent in boys than in girls during childhood [16–18], but in adolescence, asthma becomes more severe and prevalent in girls [19, 20]. This shift is attributed to changes in hormonal milieu or environmental exposures [21–23]. In line with this, the rate of asthma related hospitalization was higher among young boys and middle-aged women in this study, supporting the theory that asthma is modulated by sex hormones. Additionally, the rate of respiratory failure was found to be lower among older women compared to men. Such improvement can be attributed to menopause and supports the reversal of the well-described gender switch that occurs at puberty [7].

The role of age on asthma related health cost is important. While less than 10% of asthmatics have severe disease, this group accounts for more than 50% of all asthma-related total health care costs[24–26]. Conversely, as the world is aging, the number of people older than 60 years is projected to double between 2007 and 2050 [27]. This will create a significant burden on the healthcare system and society worldwide. As such, investigating preventable determinants of lung aging that contribute to severe asthma will become clinically and economically vital. This study provides a comprehensive analysis of hospitalization cost while taking into account the age-gender interaction and adjusting for race and age related comorbidities. The findings that asthma is more severe and expensive with children and older adults, suggest that an improved approach to asthma therapy is needed. For example, managing tobacco addiction, obesity, GERD and other age related comorbidities will be increasingly important. While mean hospitalization cost is important for individual patients, aggregate hospital cost is paramount to society and national healthcare policymakers. Our data help to identify high risk groups, such as young boys and middle age women, who can be targeted for close outpatient monitoring, asthma education and aggressive medical therapy.

HCUP databases provide a unique opportunity to analyze hospital mortality and cost. While Medicare and Medicaid databases provide accurate information regarding senior and non-privileged participants, HCUP-NIS samples all individuals admitted to a hospital in the US. This helps lowering the selection bias. Furthermore, for a disease with a very low mortality such as asthma (0.3% per hospitalization) [28], large databases are needed to detect any differences in mortality risk factors. On the other hand, HCUP does not provide any clinical or medication data, and therefore may be subject to biased results. In this context, secondary databases run the risk of not measuring all the necessary variables and risk factors [29] or missing and misclassifying important measurements [30]. Therefore, the accuracy of administrative data depend on the correct identification and documentation of the disease in the patient chart, a task that is not consistently fulfilled by clinicians [31, 32]. For example, while GERD may be under-reported in HCUP, the primary outcomes listed here are not influenced by the clinician. Mortality, hospital length of stay and hospital cost are well recorded and accurately reported to HCUP. Furthermore, in order to increase the Diagnosis-Related Group (DRG) and hospital charges, the diagnosis of respiratory failure is also less likely to be under reported. Even taking these limitations into account, observational databases continue to be useful in making accurate predictions for individual patients [33]. Importantly, the specificity of disease ascertainment through administrative claim-based data was reported to be substantially higher than 90% for most conditions but sensitivities is usually reported below 60% [34]. This fact supports the accuracy of the diagnosis of asthma in the HCUP database.

In order to minimize bias, we have provided a robust description of the data and diagnostic codes used, focusing on the sampling methods and the inclusion and exclusion criteria. We also took into consideration the unique characteristics of the database, how it was generated and the variables it measures [35, 36]. HCUP is an administrative claim-based database. The data are limited by a lack of clinical information, including physiological, radiological or laboratory testing results. Additionally, no information is available regarding medication use or compliance. Therefore it was not possible to use standard definitions to define asthma severity such as the one recommended by the American Thoracic Society Workshop on Refractory Asthma [15], or to use information on asthma related quality of life such as the one provided using the Asthma Quality of Life Questionnaire (AQLQ) [37]. To address such limitations, other markers of asthma severity, such as rate of asthma related hospitalization, hospital mortality, hospital length of stay, monetary cost and the rate of respiratory failure were used as indicators for severe asthma. Additionally, we used the NIS 2012 to fit our models and the NIS 2011 database to validate them. Most importantly, in order to externally validate and confirm the results we used the NHLBI SARP database which recruited patients over a 10 years period using strictly defined criteria for severe asthma. The analysis duplication and results validation provide a measure of reassurance that the findings are robust and true across all databases.

In summary, asthma is most severe in young males and middle aged women. In these populations, it results in the highest hospitalization rates, mortality, respiratory failure, and aggregate cost. A global multisystem approach to the health of the young and the elderly is needed in order to improve asthma control and lower cost. Moreover, we report for the first time that asthma severity decreases dramatically, though transiently, in young adulthood. Understanding the determinants of this decrease may prove important clues to help flatten the severe asthma risk curve across the entire age spectrum.

## Supporting Information

S1 Appendix ICD-9 CM Codes used in the analysis. (DOCX)

S1 Fig Conceptual framework. (DOCX)

S2 Fig Patient selection for assessment of asthma related hospital outcome. (DOCX)

S3 Fig Distribution of asthma related hospitalizations stratified by gender. (DOCX)

S4 Fig Density plot of age distribution stratified by gender using of patient with severe asthma (Panel C) or hospitalized from asthma during the previous year (Panel D) using the Severe Asthma Research Program (SARP I&II).
(DOCX)

S5 Fig Plot of the probably of asthma related respiratory failure as a function of age and stratified by gender using NIS data. (DOCX)

S6 Fig Distribution of asthma related hospital mortality stratified by gender. (DOCX)

S7 Fig Mean and aggregate hospital cost as a function of age in 2012. (DOCX)

S1 Table Data Elements in the 2011 and 2012 NIS Disease Severity Measures Files. (DOCX)

S2 Table National Statistics of Asthma Related ED visit and Hospitalizations. (DOCX)

S3 Table Estimated hospital cost and charges by gender and respiratory failure abstracted from the NIS databases. (DOCX)

S4 Table Estimated hospital cost and charges by gender and age category abstracted from the NIS databases. (DOCX)

S5 Table Estimated hospital cost and charges by gender and respiratory failure abstracted from the NIS databases. (DOCX)
