# Supplementary material for: Impact of Age and Sex on Outcomes and Hospital Cost of Acute Asthma in the United States, 2011-2012
Source: PLoS One. 2016 Jun 13;11(6):e0157301. doi: 10.1371/journal.pone.0157301 (PMC4905648; doi:10.1371/journal.pone.0157301)
Supplement: S1 Appendix — (DOCX) [file pone.0157301.s001.docx]

**S1 Appendix. ICD-9 CM Codes used in the analysis**

|  |  | **ICD-9 CM code** |
| --- | --- | --- |
| **Asthma Diagnosis:** |  |  |
|  | Extrinsic asthma unspecified: | [493.00](http://www.cms.gov/medicare-coverage-database/staticpages/icd-9-code-lookup.aspx?KeyWord=asthma&bc=AAAAAAAAAAAEAA%3d%3d&) |
|  | Extrinsic asthma with status asthmaticus | [493.01](http://www.cms.gov/medicare-coverage-database/staticpages/icd-9-code-lookup.aspx?KeyWord=asthma&bc=AAAAAAAAAAAEAA%3d%3d&) |
|  | Extrinsic asthma with (acute) exacerbation: | [493.02](http://www.cms.gov/medicare-coverage-database/staticpages/icd-9-code-lookup.aspx?KeyWord=asthma&bc=AAAAAAAAAAAEAA%3d%3d&) |
|  | Intrinsic asthma unspecified: | [493.10](http://www.cms.gov/medicare-coverage-database/staticpages/icd-9-code-lookup.aspx?KeyWord=asthma&bc=AAAAAAAAAAAEAA%3d%3d&) |
|  | Intrinsic asthma with status asthmaticus: | [493.11](http://www.cms.gov/medicare-coverage-database/staticpages/icd-9-code-lookup.aspx?KeyWord=asthma&bc=AAAAAAAAAAAEAA%3d%3d&) |
|  | Intrinsic asthma with (acute) exacerbation: | [493.12](http://www.cms.gov/medicare-coverage-database/staticpages/icd-9-code-lookup.aspx?KeyWord=asthma&bc=AAAAAAAAAAAEAA%3d%3d&) |
|  | Chronic obstructive asthma unspecified: | [493.20](http://www.cms.gov/medicare-coverage-database/staticpages/icd-9-code-lookup.aspx?KeyWord=asthma&bc=AAAAAAAAAAAEAA%3d%3d&) |
|  | Chronic obstructive asthma with status asthmaticus: | [493.21](http://www.cms.gov/medicare-coverage-database/staticpages/icd-9-code-lookup.aspx?KeyWord=asthma&bc=AAAAAAAAAAAEAA%3d%3d&) |
|  | Chronic obstructive asthma with (acute) exacerbation: | [493.22](http://www.cms.gov/medicare-coverage-database/staticpages/icd-9-code-lookup.aspx?KeyWord=asthma&bc=AAAAAAAAAAAEAA%3d%3d&) |
|  | Cough variant asthma: | [493.82](http://www.cms.gov/medicare-coverage-database/staticpages/icd-9-code-lookup.aspx?KeyWord=asthma&bc=AAAAAAAAAAAEAA%3d%3d&) |
|  | Asthma unspecified: | [493.90](http://www.cms.gov/medicare-coverage-database/staticpages/icd-9-code-lookup.aspx?KeyWord=asthma&bc=AAAAAAAAAAAEAA%3d%3d&) |
|  | Asthma unspecified type with status asthmaticu | [493.91](http://www.cms.gov/medicare-coverage-database/staticpages/icd-9-code-lookup.aspx?KeyWord=asthma&bc=AAAAAAAAAAAEAA%3d%3d&) |
|  | Asthma unspecified with (acute) exacerbation: | [493.92](http://www.cms.gov/medicare-coverage-database/staticpages/icd-9-code-lookup.aspx?KeyWord=asthma&bc=AAAAAAAAAAAEAA%3d%3d&) |
|  |  |  |
| **Gastroesophageal Reflux Disease (GERD) Diagnosis:** |  |  |
|  | Reflux esophagitis: | [530.11](http://www.cms.gov/medicare-coverage-database/staticpages/icd-9-code-lookup.aspx?KeyWord=530&bc=AAAAAAAAAAAEAA%3d%3d&) |
|  | Esophageal reflux: | [530.81](http://www.cms.gov/medicare-coverage-database/staticpages/icd-9-code-lookup.aspx?KeyWord=530&bc=AAAAAAAAAAAEAA%3d%3d&) |
| **Respiratory Syncytial Virus (RSV)** **Related Bronchiolitis Diagnosis.** |  |  |
|  | Respiratory syncytial virus: | [079.6](http://www.cms.gov/medicare-coverage-database/staticpages/icd-9-code-lookup.aspx?KeyWord=rsv&bc=AAAAAAAAAAAEAA%3d%3d&) |
|  | Acute bronchiolitis due to respiratory syncytial virus: | [466.11](http://www.cms.gov/medicare-coverage-database/staticpages/icd-9-code-lookup.aspx?KeyWord=rsv&bc=AAAAAAAAAAAEAA%3d%3d&) |
| **Chronic Obstructive Pulmonary Disease (COPD):** |  |  |
|  | Emphysematous bleb | [492.0](http://www.cms.gov/medicare-coverage-database/staticpages/icd-9-code-lookup.aspx?KeyWord=emphysema&bc=AAAAAAAAAAAEAA%3d%3d&) |
|  | Other emphysema | [492.8](http://www.cms.gov/medicare-coverage-database/staticpages/icd-9-code-lookup.aspx?KeyWord=emphysema&bc=AAAAAAAAAAAEAA%3d%3d&) |
|  | Chronic airway obstruction not elsewhere classified | [496](http://www.cms.gov/medicare-coverage-database/staticpages/icd-9-code-lookup.aspx?KeyWord=Chronic%20airway%20obstruction&bc=AAAAAAAAAAAEAA%3d%3d&) |
|  | Obstructive chronic bronchitis without exacerbation | [491.20](http://www.cms.gov/medicare-coverage-database/staticpages/icd-9-code-lookup.aspx?KeyWord=Obstructive%20chronic%20bronchitis&bc=AAAAAAAAAAAEAA%3d%3d&) |
|  | Obstructive chronic bronchitis with (acute) exacerbation | [491.21](http://www.cms.gov/medicare-coverage-database/staticpages/icd-9-code-lookup.aspx?KeyWord=Obstructive%20chronic%20bronchitis&bc=AAAAAAAAAAAEAA%3d%3d&) |
|  | Obstructive chronic bronchitis with acute bronchitis | [491.22](http://www.cms.gov/medicare-coverage-database/staticpages/icd-9-code-lookup.aspx?KeyWord=Obstructive%20chronic%20bronchitis&bc=AAAAAAAAAAAEAA%3d%3d&) |
| **Diagnosis of Respiratory Failure and Arrest:** |  |  |
|  | Acute respiratory failure | [518.81](http://www.cms.gov/medicare-coverage-database/staticpages/icd-9-code-lookup.aspx?KeyWord=%20Respiratory%20failure&bc=AAAAAAAAAAAEAA%3d%3d&) |
|  | Chronic respiratory failure | [518.83](http://www.cms.gov/medicare-coverage-database/staticpages/icd-9-code-lookup.aspx?KeyWord=%20Respiratory%20failure&bc=AAAAAAAAAAAEAA%3d%3d&) |
|  | Acute and chronic respiratory failure | [518.84](http://www.cms.gov/medicare-coverage-database/staticpages/icd-9-code-lookup.aspx?KeyWord=%20Respiratory%20failure&bc=AAAAAAAAAAAEAA%3d%3d&) |
|  | Respiratory arrest | [799.1](http://www.cms.gov/medicare-coverage-database/staticpages/icd-9-code-lookup.aspx?KeyWord=respiratory&bc=AAAAAAAAAAAEAA%3d%3d&) |
| **Tobacco Use Disorder** |  |  |
|  | Nondependent tobacco use disorder: | [305.1](http://www.cms.gov/medicare-coverage-database/staticpages/icd-9-code-lookup.aspx?KeyWord=tobacco&bc=AAAAAAAAAAAEAA%3d%3d&) |
|  | Personal history of tobacco use: | [v15.82](http://www.cms.gov/medicare-coverage-database/staticpages/icd-9-code-lookup.aspx?KeyWord=tobacco&bc=AAAAAAAAAAAEAA%3d%3d&) |
