# Supplementary material for: Impact of Age and Sex on Outcomes and Hospital Cost of Acute Asthma in the United States, 2011-2012
Source: PLoS One. 2016 Jun 13;11(6):e0157301. doi: 10.1371/journal.pone.0157301 (PMC4905648; doi:10.1371/journal.pone.0157301)

**S1 Fig. Conceptual framework**. Aging is associated with worse clinical outcome, such as higher hospitalization rate, respiratory failure and mortality higher age-related. This association is in part mediated by age related comorbidities such as obesity, congestive heart failure, malignancy, and gastro esophageal reflux disease which in turn result in, morbidity and mortality. Additionally, other moderating factors such as gender, race can impact asthma severity and subsequently clinical outcome.


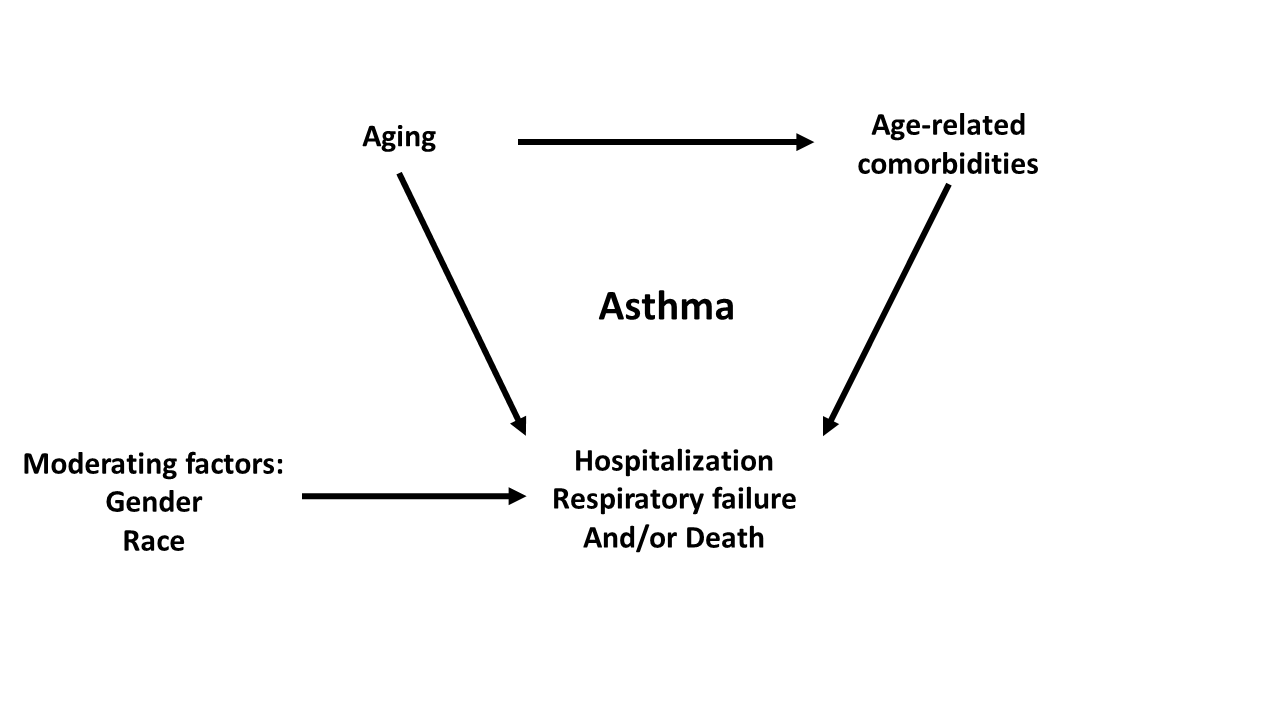

Supplement: S1 Fig — (DOCX) [file pone.0157301.s002.docx]
