# Supplementary material for: Impact of Age and Sex on Outcomes and Hospital Cost of Acute Asthma in the United States, 2011-2012
Source: PLoS One. 2016 Jun 13;11(6):e0157301. doi: 10.1371/journal.pone.0157301 (PMC4905648; doi:10.1371/journal.pone.0157301)
Supplement: S2 Fig — (DOCX) [file pone.0157301.s003.docx]

**S2 Fig. Patient selection for assessment of asthma related hospital outcome.**

**
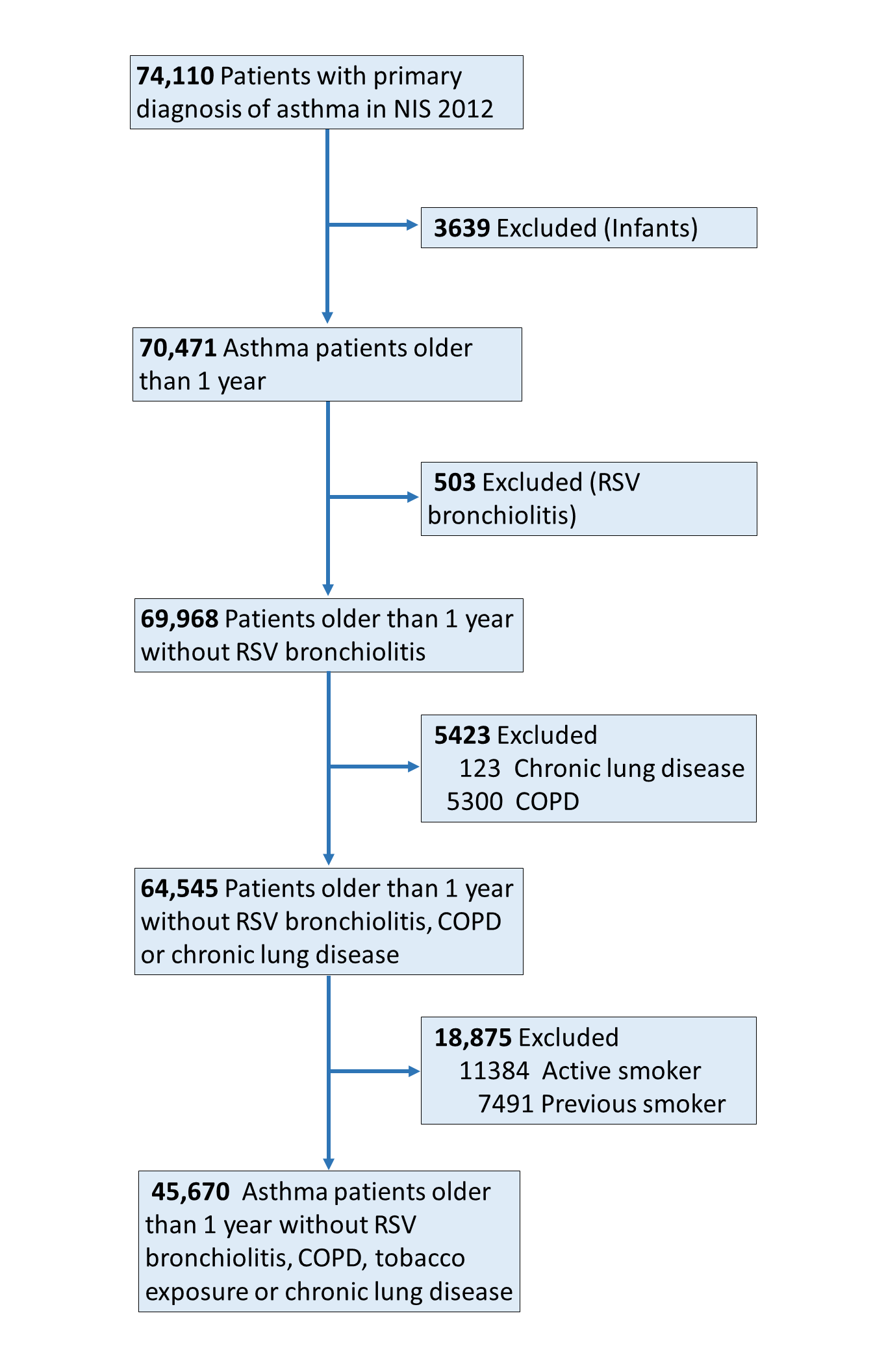
**

RSV indicates Respiratory Syncytial Virus; COPD, Chronic Obstructive Pulmonary Disease.
