# Supplementary material for: Impact of Age and Sex on Outcomes and Hospital Cost of Acute Asthma in the United States, 2011-2012
Source: PLoS One. 2016 Jun 13;11(6):e0157301. doi: 10.1371/journal.pone.0157301 (PMC4905648; doi:10.1371/journal.pone.0157301)

**S3 Fig. Distribution of asthma related hospitalizations stratified by gender**. Panels A and B reflect hospitalizations in 2011 and 2012 respectively.


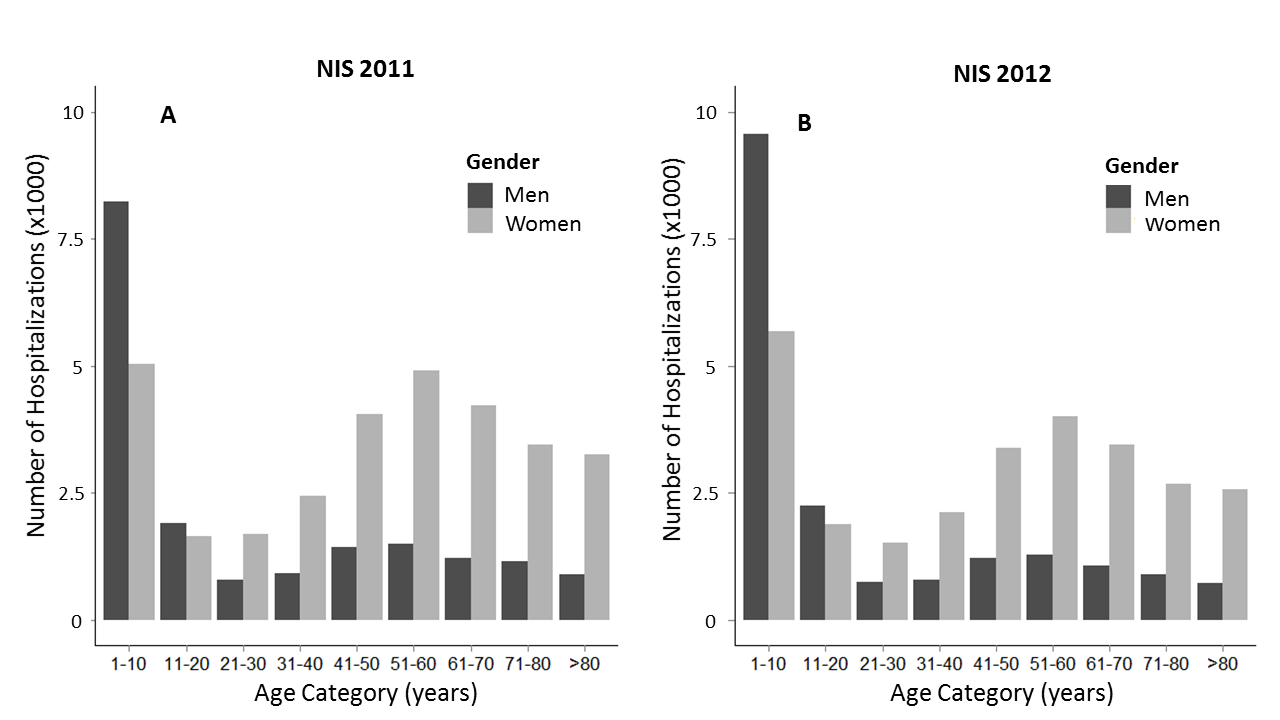

Supplement: S3 Fig — (DOCX) [file pone.0157301.s004.docx]
