# Supplementary material for: Impact of Age and Sex on Outcomes and Hospital Cost of Acute Asthma in the United States, 2011-2012
Source: PLoS One. 2016 Jun 13;11(6):e0157301. doi: 10.1371/journal.pone.0157301 (PMC4905648; doi:10.1371/journal.pone.0157301)
Supplement: S4 Fig — Density plot of age distribution stratified by gender using of patient with severe asthma (Panel C) or hospitalized from asthma during the previous year (Panel D) using the Severe Asthma Research Program (SARP I&II). (DOCX) [file pone.0157301.s005.docx]

**S4 Fig. Density plot of age distribution stratified by gender using of patient with severe asthma (Panel C) or hospitalized from asthma during the previous year (Panel D) using the Severe Asthma Research Program (SARP I&II).** The attenuation of early peak is SARP I&II could be related to the fact that SARP did not recruit children younger than 6 years.

**
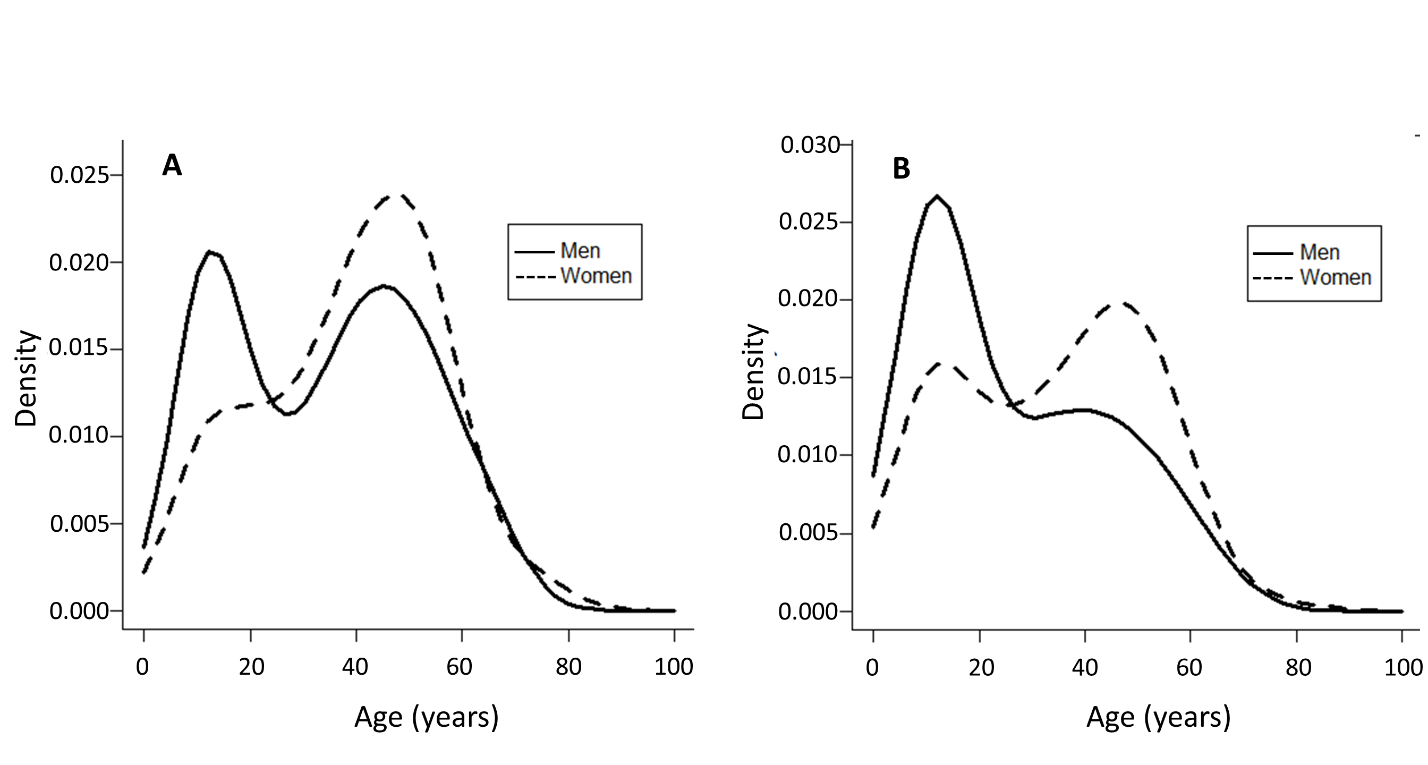
**
