# Supplementary material for: Impact of Age and Sex on Outcomes and Hospital Cost of Acute Asthma in the United States, 2011-2012
Source: PLoS One. 2016 Jun 13;11(6):e0157301. doi: 10.1371/journal.pone.0157301 (PMC4905648; doi:10.1371/journal.pone.0157301)

**S5 Fig. Plot of the probably of asthma related respiratory failure as a function of age and stratified by gender using NIS data.**

The association between age and the probability of respiratory failure is demonstrated by applying a LOWESS (Locally Weighted Scatterplot Smoother) smoother in the overall population. The relationship between age and probability of respiratory failure resembles a spline with an inflection point at the age of 60 years (Panel A). The stratification by gender shows the probability of asthma severity is higher in men than women after age 60 (Panel B).


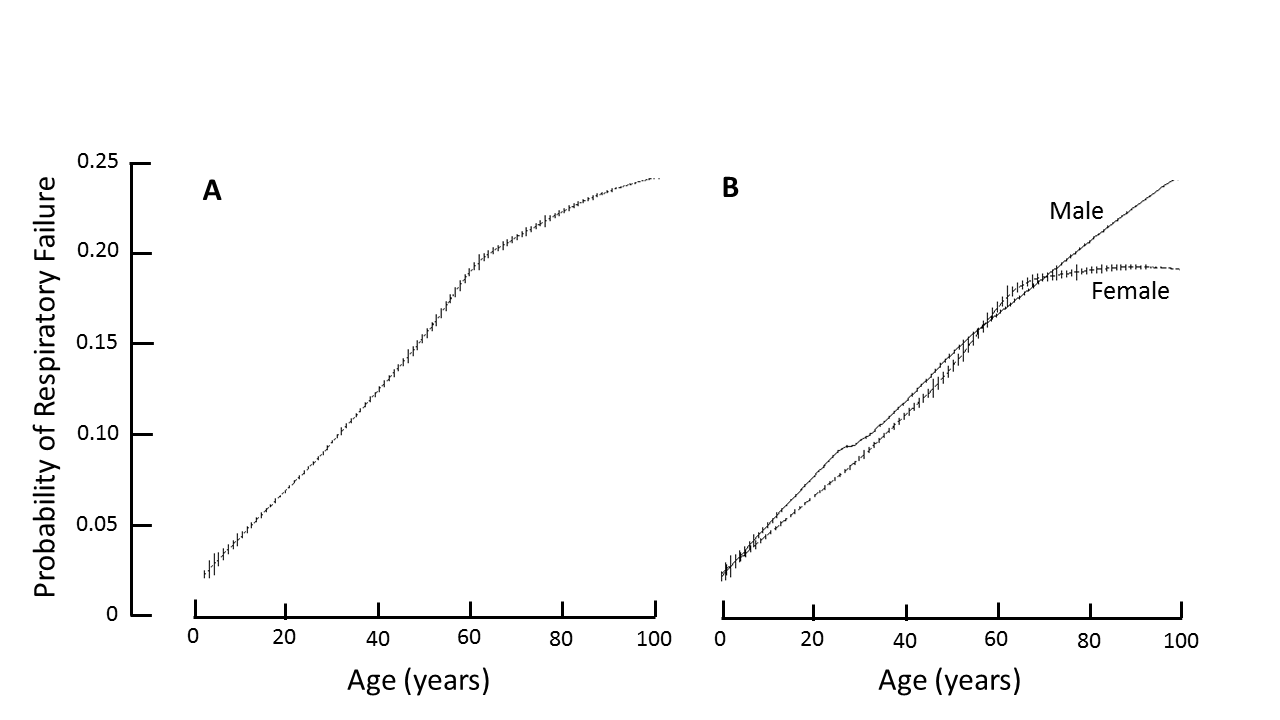

Supplement: S5 Fig — (DOCX) [file pone.0157301.s006.docx]
