# Supplementary material for: Impact of Age and Sex on Outcomes and Hospital Cost of Acute Asthma in the United States, 2011-2012
Source: PLoS One. 2016 Jun 13;11(6):e0157301. doi: 10.1371/journal.pone.0157301 (PMC4905648; doi:10.1371/journal.pone.0157301)

**S6 Fig. Distribution of asthma related hospital mortality stratified by gender.** Panels A: The number of deaths from asthma as a function of age stratified by gender. Panel B: reflect the frequency of hospital mortality stratified by gender. Overall more women older than 50 years died with asthma in the hospital. Correcting for the higher hospitalization rate among older women compared to men, the difference in the frequency of asthma related hospital mortality is smaller among older men and women.


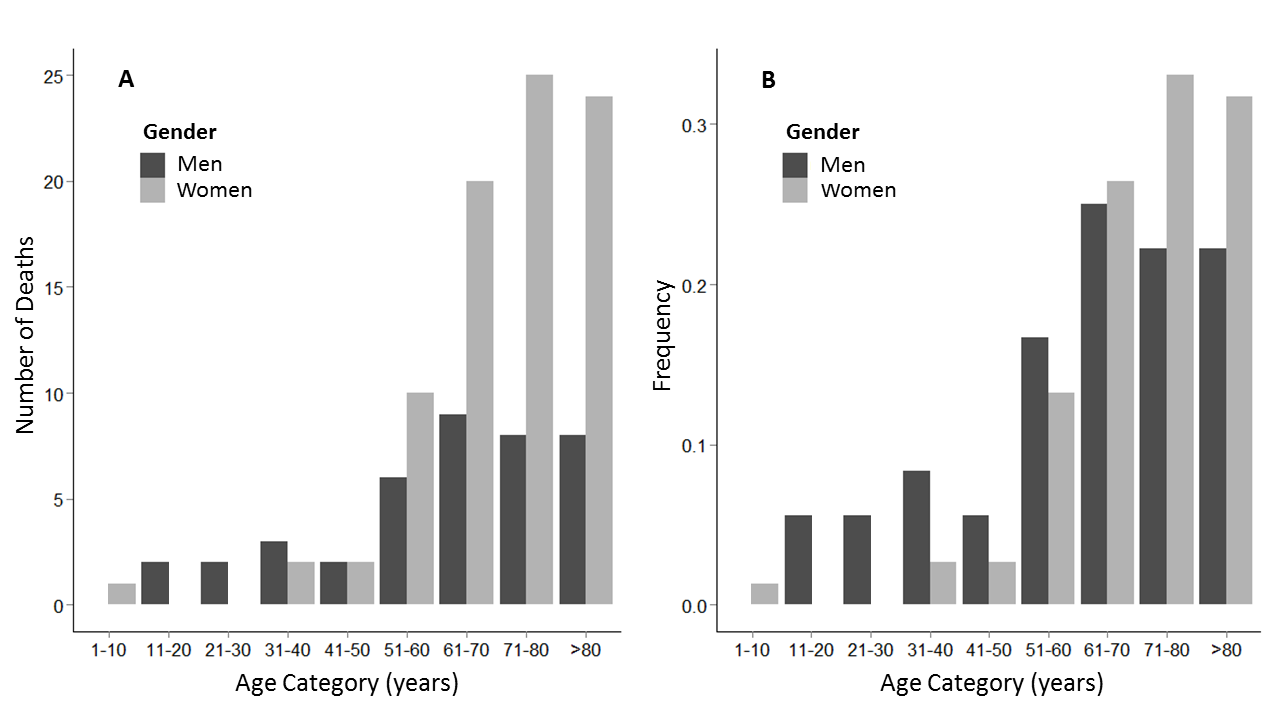

Supplement: S6 Fig — (DOCX) [file pone.0157301.s007.docx]
