# Supplementary material for: Impact of Age and Sex on Outcomes and Hospital Cost of Acute Asthma in the United States, 2011-2012
Source: PLoS One. 2016 Jun 13;11(6):e0157301. doi: 10.1371/journal.pone.0157301 (PMC4905648; doi:10.1371/journal.pone.0157301)

**S7 Fig.** **Mean and aggregate hospital cost as a function of age in 2012.** Panel A: Mean and Standard Error plot of asthma related hospitalization cost across age categories stratified by gender showing than mean hospitalization cost increases with age and is higher in women compared to men. Panel B reflect the aggregate hospital cost across age categories also showing the bimodal distribution of aggregate cost. Panel A and B are abstracted from NIS 2012.


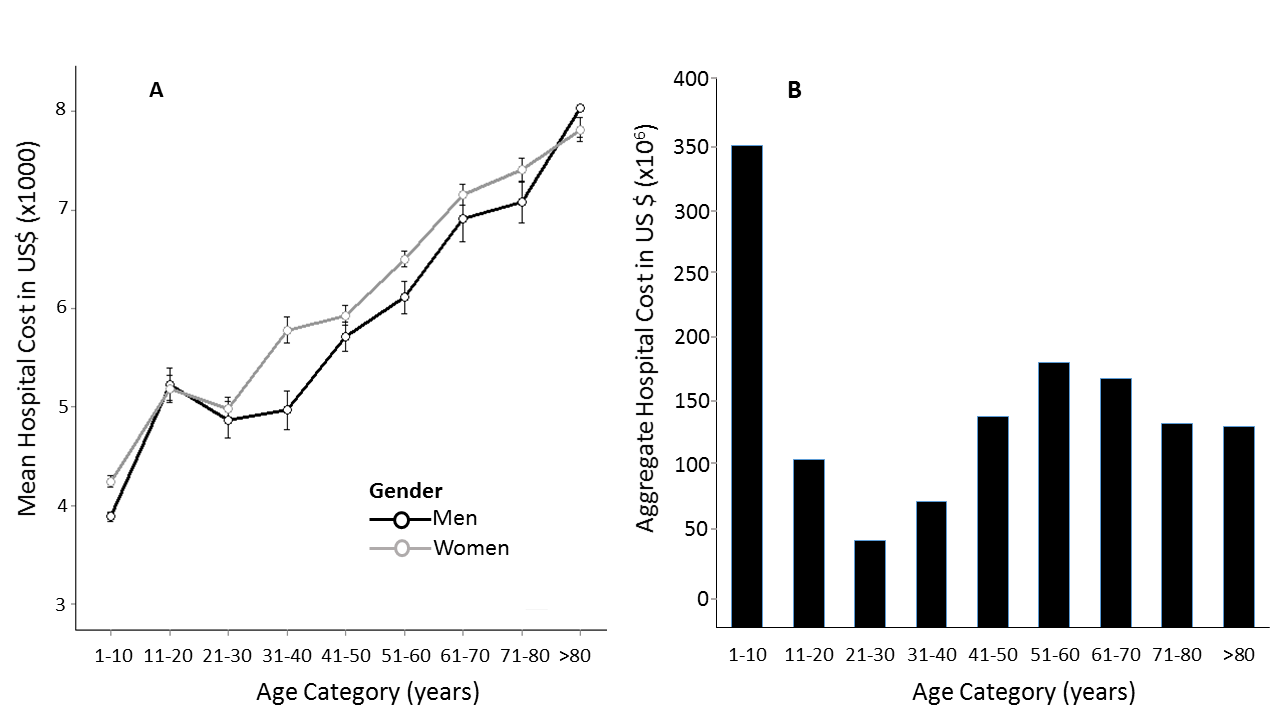

Supplement: S7 Fig — (DOCX) [file pone.0157301.s008.docx]
