# Supplementary material for: Impact of Age and Sex on Outcomes and Hospital Cost of Acute Asthma in the United States, 2011-2012
Source: PLoS One. 2016 Jun 13;11(6):e0157301. doi: 10.1371/journal.pone.0157301 (PMC4905648; doi:10.1371/journal.pone.0157301)
Supplement: S1 Table — (DOCX) [file pone.0157301.s009.docx]

**S1 Table. Data Elements in the 2011 and 2012 NIS Disease Severity Measures Files.**

| **Comorbidities** | **HCUP Code** |
| --- | --- |
| Acquired Immunodeficiency Syndrome (AIDS) | CM_AIDS |
| Alcohol abuse | CM_ALCOHOL |
| Deficiency anemia | CM_ANEMDEF |
| Rheumatoid Arthritis/Collagen Vascular Disease (RA/CTD) | CM_ARTH |
| Blood loss | CM_BLDLOSS |
| Congestive Heart Failure (CHF) | CM_CHF |
| Chronic lung disease* | CM_CHRNLUNG |
| Coagulopathy | CM_COAG |
| Depression | CM_DEPRESS |
| Diabetes (DM) | CM_DM |
| Diabetes with Chronic complications | CM_DMCX |
| Drug abuse | CM_DRUG |
| Hypertension (combine uncomplicated and complicated) | CM_HTN_C |
| Hypothyroidism | CM_HYPOTHY |
| Liver disease | CM_LIVER |
| Lymphoma | CM_LYMPH |
| Fluid and electrolyte disorders | CM_LYTES |
| Metastatic cancer | CM_METS |
| Other neurological disorders | CM_NEURO |
| Obesity | CM_OBESE |
| Paralysis | CM_PARA |
| Peripheral vascular Disorders | CM_PERIVASC |
| Psychosis | CM_PSYCH |
| Pulmonary circulation disorder | CM_PULMCIRC |
| Renal failure | CM_RENLFAIL |
| Solid tumor without metastasis | CM_TUMOR |
| Peptic ulcer disease excluding bleeding | CM_ULCER |
| Valvular heart disease (VHD) | CM_VALVE |
| Weight loss | CM_WGHTLOSS |

*patients with chronic lung diseases were excluded from this analysis as detailed in the method section of the manuscript.
