# Supplementary material for: Impact of Age and Sex on Outcomes and Hospital Cost of Acute Asthma in the United States, 2011-2012
Source: PLoS One. 2016 Jun 13;11(6):e0157301. doi: 10.1371/journal.pone.0157301 (PMC4905648; doi:10.1371/journal.pone.0157301)
Supplement: S2 Table — (DOCX) [file pone.0157301.s010.docx]

**S2 Table. National Statistics on Asthma Hospitalizations.**

|  | **2011** | **2012** |
| --- | --- | --- |
| Total number of hospitalization | 368,528 | 372,685 |
| Female gender (%) | 232,970 (63) | 226,915 (61) |
| Length of Stay , days (mean) | 3.4 | 3.2 |
| Length of stay, days (median) | 3.0 | 2.0 |
| Charges, $ (mean) | 20,756 | 21,512 |
| Charges, $ (median) | 13,926 | 14,350 |
| Cost, $ (mean) | 5,907 | 5,981 |
| Cost, $ (median) | 4,336 | 4,380 |
| Aggregate Charges, $ | 7,649,167,168 | 8,017,199,720 |
| Aggregate Cost, $ | 2,176,015,890 | 2,231,112,607 |
| In-Hospital Deaths | 1,088 (0.30%) | 1,090 (0.29%) |
