# Supplementary material for: Impact of Age and Sex on Outcomes and Hospital Cost of Acute Asthma in the United States, 2011-2012
Source: PLoS One. 2016 Jun 13;11(6):e0157301. doi: 10.1371/journal.pone.0157301 (PMC4905648; doi:10.1371/journal.pone.0157301)
Supplement: S3 Table — (DOCX) [file pone.0157301.s011.docx]

**S3 Table. Racial differences in mean age among adult 18 years and older hospitalized in 2012.**

|  | **Mean difference**  **(Years)** | **95% CI** | **p value** |
| --- | --- | --- | --- |
| African American vs. White | -8.65 | -9.36; -7.93 | <0.000 |
| Hispanic vs. White | -4.80 | -5.71; -3.88 | <0.000 |
| Asians or Pacific Islander vs. White | 5.15 | 3.15; 7.15 | <0.000 |
| Native American vs. White | -4.33 | -8.12; -0.54 | <0.016 |
| Hispanic vs. African American | 3.85 | 2.87; 4.84 | <0.000 |
| Asian or Pacific Islander vs. African American | 13.80 | 11.76; 15.83 | <0.000 |
| Native American vs. African American | 4.32 | 0.51; 8.13 | <0.017 |
| Asian or Pacific Islander vs. Hispanic | 9.94 | 7.83; 12.05 | <0.000 |
| Native American vs. Hispanic | 0.47 | -3.38; 4.32 | 0.997 |
| Native American vs. Asian or Pacific Islander | -9.47 | -9.36; -7.93 | <0.000 |
