# Supplementary material for: Impact of Age and Sex on Outcomes and Hospital Cost of Acute Asthma in the United States, 2011-2012
Source: PLoS One. 2016 Jun 13;11(6):e0157301. doi: 10.1371/journal.pone.0157301 (PMC4905648; doi:10.1371/journal.pone.0157301)
Supplement: S4 Table — (DOCX) [file pone.0157301.s012.docx]

**S4 Table. Estimated hospital cost and charges by gender and age category abstracted from the NIS databases.**

|  |  | **Total Charges ($)** | | **Total Cost ($)** | |
| --- | --- | --- | --- | --- | --- |
| **NIS**  **data** | **Age (years)** | **Men** | **Women** | **Men** | **Women** |
| 2012 |  |  |  |  |  |
|  | <1 | 8653 [5447-14456] | 8871 [5363-14903] | 2655 [1722-4212] | 2653 [1768-4243] |
|  | 1-10 | 9475 [5796-15486] | 10106 [6141-16617] | 2823 [1886-4528] | 3009 [1991-4898] |
|  | 11-20 | 11579 [6829-19978] | 11830 [7111-20876] | 3454 [2234-5837] | 3584 [2305-5851] |
|  | 21-30 | 11542 [7038-18381] | 13190 [7959-21402] | 3501 [2394-5291] | 3891 [2634-5909] |
|  | 31-40 | 13276 [8139-21318] | 14273 [8511-23755] | 3879 [2629-5929] | 4284 [2879-6595] |
|  | 41-50 | 15188 [8982-25313] | 15898 [9598-26322] | 4504 [3022-6877] | 4763 [3188-7188] |
|  | 51-60 | 15804 [9627-26936] | 17951 [10741-30403] | 4857 [3265-7290] | 5345 [3544-8036] |
|  | 61-70 | 18301 [11269-30768] | 19259 [11469-31798] | 5414 [3729-8288] | 5774 [3868-8755] |
|  | 71-80 | 18880 [11610-31619] | 20108 [11974-34609] | 5497 [3826-8578] | 5978 [4002-9223] |
|  | >80 | 20543 [12038-35976] | 20862 [12441-35757] | 5982 [4011-9497] | 6229 [4241-9487] |
| 2011 |  |  |  |  |  |
|  | <1 | 7840 [4912-12870] | 8062 [5156-13709] | 2514 [1679-3899] | 2555 [1662-4130] |
|  | 1-10 | 8411 [5399-13320] | 8912 [5529-14841] | 2616 [1792-4042] | 2881 [1942-4538] |
|  | 11-20 | 10280 [6482-17553] | 10465 [6526-17233] | 3315 [2190-5152] | 3359 [2257-5232] |
|  | 21-30 | 10959 [6977-18216] | 12293 [7584-20324] | 3419 [2292-5330] | 3710 [2562-5630] |
|  | 31-40 | 13055 [8051-21901] | 13492 [8380-22235] | 3836 [2671-5957] | 4136 [2814-6376] |
|  | 41-50 | 14095 [8854-23847] | 15418 [9430-26208] | 4353 [2931-6455] | 4686 [3133-7183] |
|  | 51-60 | 17011 [10064-27732] | 16958 [10166-29113] | 5042 [3323-7536] | 5191 [3484-7958] |
|  | 61-70 | 18022 [10644-30174] | 18570 [11003-30943] | 5446 [3609-8348] | 5694 [3770-8670] |
|  | 71-80 | 19171 [11187-32253] | 20051 [11967-34575] | 5815 [3962-8761] | 5987 [4027-9137] |
|  | >80 | 20139 [11446-33970] | 20120 [12054-34314] | 6140 [4080-9192] | 6129 [4140-9381] |

Results are reported as median and Interquartile Range (median[IQR])
