# Supplementary material for: Impact of Age and Sex on Outcomes and Hospital Cost of Acute Asthma in the United States, 2011-2012
Source: PLoS One. 2016 Jun 13;11(6):e0157301. doi: 10.1371/journal.pone.0157301 (PMC4905648; doi:10.1371/journal.pone.0157301)
Supplement: S5 Table — (DOCX) [file pone.0157301.s013.docx]

**S5 Table. Estimated hospital cost and charges by gender and respiratory failure abstracted from the NIS databases.**

|  |  | **Total Charges ($)** | | **Total Cost ($)** | |
| --- | --- | --- | --- | --- | --- |
| **NIS data** | **Outcome** | **Men** | **Women** | **Men** | **Women** |
| 2012 |  |  |  |  |  |
|  | No Respiratory Failure | 11708 [6945-20089] | 25319 [14848-41178] | 3570 [2271-5711] | 4499 [2907-6998] |
|  | Respiratory  Failure | 19651 [11387-36304] | 40305 [22557-72471] | 6193 [3882-10455] | 6926 [4442-11318] |
|  | Requiring Mechanical Ventilation | 59422 [31795-109338] | 62423 [37565-108267] | 17176 [10312-32479] | 18619 [11306-28369] |
| 2011 |  |  |  |  |  |
|  | No Respiratory Failure | 11262 [6754-19514] | 14537 [8647-25094] | 3522 [2248-5659] | 4515 [2915-7044] |
|  | Respiratory  Failure | 21040 [11557-40131] | 22212 [12816-41108] | 6334 [3853-10994] | 6700 [4314-11409] |
|  | Requiring Mechanical Ventilation | 67888 [36915- 127068] | 69643 [41251-127368] | 19482 [10818-33630] | 21029 [12531-36749] |

Results are reported as median and Interquartile Range (median[IQR])
